# Open challenges for the automatic synthesis of clinical trials

**DOI:** 10.1186/s13104-025-07121-6

**Published:** 2025-02-02

**Authors:** Olivia Sanchez-Graillet, David M. Schmidt, Christian Kullik, Philipp Cimiano

**Affiliations:** 1 Semantic Computing Group, Center for Cognitive Interaction Technology, Bielefeld University, Inspiration 1, 33619 Bielefeld, NRW Germany; 2 Faculty of Technology, Bielefeld University, Universitätsstraße 25, 33615 Bielefeld, NRW Germany

**Keywords:** Clinical trial synthesis, Core outcome set, COMET taxonomy, Outcome grouping

## Abstract

**Objective:**

An important criterion for selecting clinical trials to be compared in systematic reviews and meta-analyses is that they measure the same outcomes. However, this represents a challenge as there is a wide variety of outcomes, and it is difficult to standardize them for comparing clinical trials containing them. To address this challenge, we utilized our annotated dataset, which includes 211 abstracts of clinical trials related to glaucoma and type 2 diabetes mellitus. We then developed a tool that provides an overview of the annotated clinical trial information and enables users to group them by outcomes.

**Results:**

Using our visualization tool, we formed groups of outcomes and their respective clinical trials. We were able to determine the most common outcomes in clinical trials for these diseases. As a case study on diabetes, we compared our outcomes with those consented by diabetes stakeholders and found that many of the grouped outcomes are aligned with the consented ones. This demonstrates that tools such as the one presented can help standardize clinical outcomes, which in turn help in the synthesis of clinical trials. Finally, we also offer some recommendations that could help in the automation of clinical trials based on outcome standardization.

**Supplementary Information:**

The online version contains supplementary material available at 10.1186/s13104-025-07121-6.

## Introduction

Systematic reviews (SR) and meta-analyses (MA) are regarded as the gold standard in healthcare decision-making because they offer strong evidence for addressing clinical research questions by synthesizing clinical trial (CT) information. Therefore, they are valuable resources for clinicians, government policymakers, and pharmaceutical companies as they help determine which treatments are the best for a specific disease and population in terms of efficiency and safety. This can also help in deciding on the costs and availability of treatments. However, conducting these studies can be time-consuming and arduous, due to the many steps involved such as literature search, removal of duplicate citations, study selection, data extraction, quality assessment, statistical analysis, data visualization and report writing [1, 2]. While tools are available to assist with this process, many still require vast human intervention [2–4]. In this paper, we mainly focus on an important part of the selection stage, which consists of deciding which CT publications are eligible for a SR or MA. An important criterion is to select those CTs that have comparable outcomes that can be synthesized. However, due to the heterogeneity of outcomes and their measurements, it can be difficult to correctly compare and combine studies of the same disease. An effort to mitigate the heterogeneity problem is made by the Core Outcome Measures in Effectiveness Trials (COMET)^1^ initiative [5]. COMET is working on creating standard outcomes for each specific disease called core outcome sets (COS) [6]. This is achieved through consensus among relevant stakeholders, including patients and healthcare professionals, who agree on the minimum set of standard outcomes for a particular disease. The expectation is that researchers use COSs in all studies for a particular disease, adding other outcomes if necessary. This would facilitate the automation of SRs and improve the quality of their assessments.

In this paper, we explore the challenges that may arise when automatically synthesizing CT information. To this end, we analyze the information contained in annotated CT abstracts [7] using a web tool developed for this purpose. We assume that our analysis applies to cases where the information is already available in a format that can be automatically processed. The following sections describe the methods used for our study, a case study on T2DM, a discussion on the challenges for the automation of CT synthesis and ways towards it, and a discussion of the limitations of the present study and final remarks.

## Methods

In this section, we describe the dataset used and the developed web-based tool for our study.

### Dataset

We use an annotated corpus that consists of 107 abstracts on glaucoma and 104 abstracts on type 2 diabetes mellitus (T2DM). These abstracts were previously utilized in another study for the synthesis of CTs [8]. In this earlier work, glaucoma was selected due to its relatively well-defined outcomes, while T2DM was chosen due to the expertise of one of our medical collaborators in this area, despite it being more complex than glaucoma. The abstracts were annotated following the C-TrO ontology schema [9] that covers the PICO elements (P: population/problem, I: intervention, C: comparison, and O: outcome), which are relevant components of a CT. C-TrO considers intervention groups (or arms) to measure the outcomes/endpoints^2^ of the studied interventions/treatments, as well as other relevant elements of published clinical studies, such as baseline values, changes from baseline values caused by interventions, and the corresponding statistical information. Supplementary Fig. 1 shows the C-TrO schema, and the annotation dataset can be accessed via the link provided in “Availability of data and materials”.

### The clinical trial visualization tool (CTV tool)

**Fig. 1:**
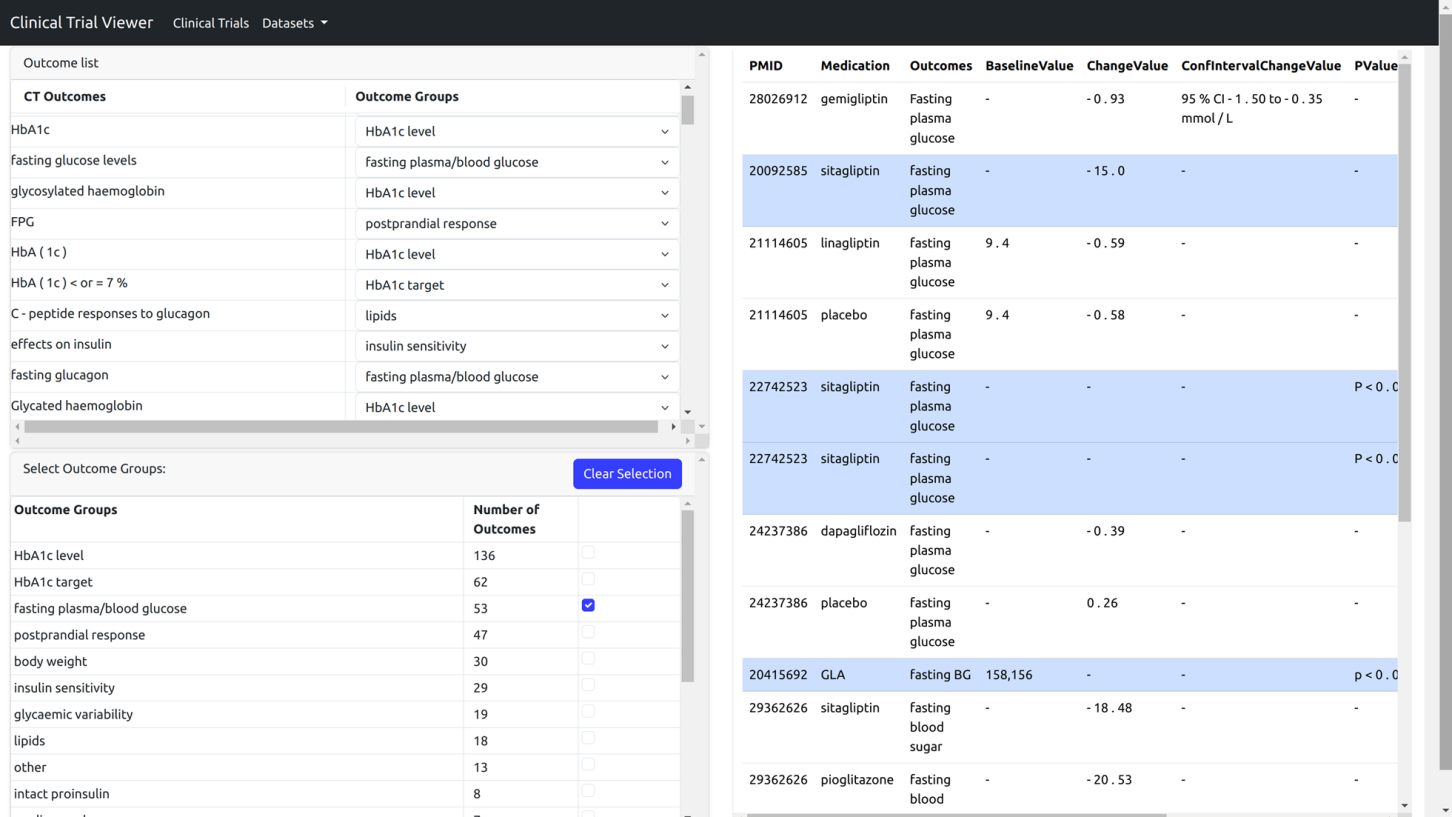
Overview interface of the CTV tool. In the example, the group “fasting plasma/blood glucose” is selected. It contains 53 CTs which are listed on the right side

Figure 1 depicts the overview interface of the web-based CTV tool we have developed for the purpose of this study.^3^ The upper left area contains a list of CT outcomes. To facilitate the assignment process, the outcomes are initially assigned to outcome groups. The initial assignment is done by calculating the similarity between an outcome and the names of the available outcome groups using the Levenshtein distance [10] which measures the similarity between two strings. The group name with the highest similarity score is then chosen to represent the outcome. Users can remove an outcome from a group by selecting “No group” in the group drop-down list. Or they can move it to another group by selecting a different group from this list. The bottom left area displays a summary of the formed groups, showing the number of outcomes in each group. Clicking on a group name displays the list of CTs containing the outcomes belonging to that group on the right side area, where detailed information about a selected CT is shown by clicking on it.

## Case study on T2DM

The goal of this case study was to see to what extent we could categorize the outcomes in our T2DM dataset according to a consented set of outcomes (i.e., COS) for T2DM. We chose to conduct the study on T2DM since, at the time of writing this paper, there was only one suitable SR identifying COS for T2DM, yet none for glaucoma in the COMET database [11]. We then chose the available SR in COMET called SCORE-IT (Selecting Core Outcomes for Randomised Effectiveness Trials in Type 2 Diabetes) [12] as the baseline for comparison. In SCORE-IT, the identification of core outcomes entailed first extracting potential core outcomes from registered trials of therapies for T2DM. Subsequently, they were categorized according to the COMET outcome domains. Discrepancies in their categorization were resolved through consensus among SCORE-IT authors. The majority of analyzed trials (88%) in SCORE-IT contain outcomes in the “metabolism and nutrition” domain. The path followed by these core outcomes in the COMET taxonomy is: *“Physiological/clinical” core area* $\rightarrow$ *“Metabolism and nutrition” core domain* $\rightarrow$ *T2DM core outcomes*. The identified core outcomes are related to lipids and lipoproteins (21%), HbA1c (18%), hypoglycaemia (14%), fasting plasma/blood glucose (11%), glycaemic variability (8%), postprandial response (8%), and insulin sensitivity (5%). The remaining outcomes are diverse.

We used the CTV tool to visualize and assign outcomes to the SCORE-IT COS (or SCORE-IT groups). When there were not any suitable SCORE-IT groups to categorize two or more similar outcomes, we created groups into which these outcomes could be categorized (i.e., potential COS) and added to the CTV tool (e.g. body weight). If there was only one outcome without a category, it was assigned to the general category “Other” (e.g. bile acid synthesis). In this way, we categorized all outcomes. Table 1 shows the outcome groups for T2DM. The first column contains the SCORE-IT COS and the second column has the outcome groups created and that are not included in SCORE-IT.

**Table 1:** Outcome groups for T2DM. (*Outcomes identified in this study and not included in SCORE-IT)

T2DM (SCORE-IT COS)	T2DM* (Not SCORE-IT COS)
Fasting plasma/blood glucose	B12 levels
Glycaemic variability	Body weight
HbA1c	Diet
Hypoglycaemia	Fat mass
Insulin sensitivity	Inflammatory cytokine
Lipids and lipoproteins	Insulinaemia
Postprandial response	Intact proinsulin
	Life quality
	Proinsulin levels
	Other

After grouping the outcomes of the CTs in our dataset, we found that similar to SCORE-IT, the domain **“metabolism and nutrition”** was the most frequent, with the following core outcomes related to HbA1c (14%), HbA1c with a given level target (30%), postprandial response (14%), fasting plasma/blood glucose (9%), insulin sensitivity (7%), glycemic variability (4%), and lipids (4%)^4^. The remaining outcomes (18%) had low percentages and were assigned to the groups in the second column of Table 1. It is important to note that in SCORE-IT the *number of CTs containing outcomes* is counted, whereas we consider *all outcomes reported in the CT abstracts* in our counts. This includes outcome subgroups and time point outcomes. Nevertheless, our counting provides a good indication of which outcomes are most commonly found in T2DM trials.

We could categorize 82% of the outcomes into the SCORE-IT groups and the rest (18%) into the groups we created. This shows that we can use SCORE-IT to a large extent and also identify new potential core outcomes with the CTV tool.

## Challenges for the automatic synthesis of clinical trials

In this section, we discuss the challenges encountered during our endeavor to automate outcome-based CT synthesis. Our analysis utilizes both glaucoma and T2DM datasets.

**Authors may describe the same outcome in different ways**. For example, Table 2 shows different descriptions for the same outcome “Diurnal IOP” across different CTs. The naming of outcomes may be ambiguous, at different levels of detail, or overly descriptive, making it uncertain whether a set of outcomes can be considered similar and therefore comparable. Furthermore, the naming of outcomes may include, for example, a target measurement (e.g., HbA(1c) < or = 7%) or any other characteristic that describes their function in a CT rather than what they intrinsically are. This lack of unique outcomes that can group outcomes that refer to the same concept in the same clinical context but with different wording can lead to varied and seemingly different outcomes across CTs [13], complicating CT synthesis.

**Table 2:** Different outcome descriptions for “Diurnal IOP”

Description	
IOP during the diurnal period	
daytime IOP	
diurnal intraocular pressure	
diurnal (in the context of IOP in a glaucoma CT)	

Authors often report their methods and results **without following recommendations for writing abstracts** that despite being short, accurately describe the relevant CT information. This often results in missing relevant CT information from abstracts. For example, the Consolidated Standards of Reporting Clinical Trials (CONSORT) [14] outlines recommendations for reporting clinical trial abstracts and outcomes in trial reports [15, 16]. However, in many cases, publishers and CT registries do not require authors to use standard results and follow reporting guidelines such as CONSORT.

**Developing a COS** can be a slow and hard process since it requires reaching a consensus among relevant stakeholders. Besides the ambiguity and inconsistency in how outcomes are described across different studies, a major obstacle, as noted by Saldanha et al. [17], can be matching the scope of the COS with the scope of the SR. For example, the COS *target population* may be too narrow/broad compared to the population in the SR, or the COS *target intervention* may be too narrow/broad relative to the SR intervention [17]. Thus, it is not a general practice for researchers to use a COS in CT protocols and SRs.

**Grouping CTs by duration or time points** can be confusing, as the same treatments across CTs may have different measurement values depending on when the measurements were taken.

**The heterogeneity of measurements** has to be considered when synthesizing CTs or pooling them for secondary studies. For example, clinicians should consider converting the measurement values to homogenized outcome information for SRs when similar outcomes across CTs are reported in different units. However, pooling is only feasible if there is highly detailed information about the original studies, and collaboration with the original authors of the CTs, who can retain the ownership of their data. Other challenges in combining data are the quality and completeness of the data being shared, different ways of reporting events, different designs, and insufficient description of the trial settings. These factors can lead to misinterpretations or bias. In this respect, Wilkinson et al. [18] observed that if the protocols of different trials are not identical, but their baseline populations are similar enough, then their data can be integrated and combined.

The analysis of the CT information and its grouping based on core outcomes shows that several aspects of automatic CT synthesis still need to be addressed. In the following section, we offer some suggestions that we believe could contribute to the automation of CT synthesis.

## The way towards the automation of clinical trials synthesis

To advance in the automation of CT synthesis, we propose the following:

1. Journals and CT registries should require the use of standard outcomes, like COSs and the COMET taxonomy, when reporting CTs. If standard outcomes are not used, authors should explain their reasons, providing valuable feedback to initiatives that develop such standard outcomes.
2. Journals and CT authors should adhere to guidelines such as CONSORT when writing abstracts.
3. Standard outcome sets should be defined by identifying common outcomes across trials studying the same disease and creating a consented list of them. This can be done by experts in the specific medical area, supported by tools such as the one presented in this work.
4. Standardized outcomes should be transformed into DOI-type objects, which have persistent identifiers. This object could contain information such as a full description, provenance, synonyms of the outcome name in a vocabulary, etc. In this way, such *“digital outcome identifiers”* could be linked to other clinical information available on the web through knowledge graphs and specialized vocabularies.
5. Tools should be used to enable the input of CT information into a structured template, following an ontology or knowledge graph structure, to ensure that the information is machine-readable (e.g., CTrO-Editor [19]).

Adopting these suggestions would improve and facilitate searching, synthesis of CTs, and reuse of CTs in secondary studies. These measures are aligned with the FAIR principles [20] to make CT data machine-readable, enabling computer systems to find, access, interoperate, and reuse CT data. This approach would also help address the growing volume of CT information and the ambiguity surrounding outcome descriptions.

## Limitations and final remarks

Currently, there are very few SRs of core outcomes for most medical conditions in the COMET database or other sources. This makes it challenging to compare outcomes from trials of different conditions using consented core outcomes. Since our dataset only covers two health conditions, it also limits our ability to compare diseases for which core outcomes are available. Some CT information may be missing in our dataset because CT abstracts may not contain all relevant information or due to missed/wrong assigned annotations. Despite these limitations, it has been demonstrated that tools such as the CTV tool can help to identify and categorize common outcomes, which may contribute to future efforts in developing core outcomes for diseases which do not have them yet. This can potentially enhance the automation of CT synthesis, as well as their search and reuse.

## Supplementary Information

Supplementary Material 1: Supplementary Fig. 1 shows the schema containing the main classes and properties of the C-TrO ontology. This schema was used for the annotation of the CT abstracts, which were used as the dataset for the present study.

## Data Availability

The annotated dataset used during the current study is available in the Semantic Computing Group at Bielefeld University repository, https://github.com/ag-sc/CT-Corpus The CTV tool can be accessed at https://ag-sc.techfak.uni-bielefeld.de/ctvis/endpoints.html

## Abbreviations

COMET: Core outcome measures in effectiveness trials
CT: Clinical trial
COS: Core outcome set
CTV: Clinical trial visualization
DOI: Digital object identifier
CONSORT: Consolidated standards of reporting clinical trials
FAIR: Findability, accessibility, interoperability, and reusability
MA: Meta-analysis
SR: Systematic review
T2DM: Type 2 diabetes mellitus

## Footnotes

1 COMET is funded by public institutions such as the European Commission.

2 The term “endpoint” in CTs refers to targeted outcomes which are statistically analyzed.

3 The link to the CVT tool is provided in “Availability of data and materials”.

4 *Hypoglycemia* is annotated in the dataset as an adverse effect, so it is not included in our outcome list.

## References

[1] Tsafnat G, Glasziou P, Choong MK, Dunn A, Galgani F, Coiera E. Systematic review automation technologies. Syst Rev. 2014;3:1–15. 10.1186/2046-4053-3-74. 25005128 10.1186/2046-4053-3-74 PMC4100748

[2] Kohl C, McIntosh EJ, Unger S, et al. Online tools supporting the conduct and reporting of systematic reviews and systematic maps: a case study on cadima and review of existing tools. Environ Evid. 2018;7(1):8. 10.1186/s13750-018-0115-5.

[3] Wu W, Akers K, Hu E, et al. Digital tools for managing different steps of the systematic review process (poster). In: The Medical Library Association Annual Meeting (2018). https://digitalcommons.wayne.edu/libsp/136.

[4] Babineau J. Product review: covidence (systematic review software). J Can Health Lib Assoc/Journal de l’Association des bibliothèques de la santé du Canada. 2014;35(2):68–71. 10.5596/c14-016.

[5] COMET. The COMET Initiative. https://www.comet-initiative.org/Resources/OutcomeClassification. Accessed: 17 June 2024.

[6] Kirkham JJ, Williamson P. Core outcome sets in medical research. BMJ Med. 2022. 10.1136/bmjmed-2022-000284. 36936568 10.1136/bmjmed-2022-000284 PMC9951367

[7] Sanchez Graillet O, Witte C, Grimm F, Cimiano P. An annotated corpus of clinical trial publications supporting schema-based relational information extraction. J Biomed Semant. 2022. 10.1186/s13326-022-00271-7. 10.1186/s13326-022-00271-7 PMC9128209 35606797

[8] Sanchez-Graillet O, Witte C, Grimm F, Grautoff S, Ell B, Cimiano P. Synthesizing evidence from clinical trials with dynamic interactive argument trees. J Biomed Semant. 2022;13(1):16. 10.1186/s13326-022-00270-8. 10.1186/s13326-022-00270-8 PMC9166347 35659056

[9] Sanchez-Graillet O, Cimiano P, Witte C, Ell B. C-TrO: an ontology for summarization and aggregation of the level of evidence in clinical trials. In: Proc. of the 5th Joint Ontology Workshops (JOWO): Ontologies and Data in the Life Sciences, Graz, Austria. 2019. http://ceur-ws.org/Vol-2518/paper-ODLS7.pdf.

[10] Levenshtein VI. Binary codes capable of correcting deletions. Insertions and Reversals Soviet Physics Doklady. 1966;10(8):707–10.

[11] COMET. The COMET Database. https://www.comet-initiative.org/Studies. Accessed: 17 June 2024.

[12] Harman NL, James R, Wilding J, Williamson PR. SCORE-IT (Selecting Core Outcomes for Randomised Effectiveness trials In Type 2 diabetes): a systematic review of registered trials. Trials. 2017;18:1–13. 10.1186/s13063-017-2317-5. 29246177 10.1186/s13063-017-2317-5 PMC5732470

[13] Young AE, Brookes ST, Avery KN, Davies A, Metcalfe C, Blazeby JM. A systematic review of core outcome set development studies demonstrates difficulties in defining unique outcomes. J Clin Epidemiol. 2019;115:14–24. 10.1016/j.jclinepi.2019.06.016. 31276780 10.1016/j.jclinepi.2019.06.016

[14] Hopewell S, Clarke M, Moher D, et al. CONSORT for reporting randomized controlled trials in journal and conference abstracts: explanation and elaboration. PLoS Med. 2008;5(1):20. 10.1371/journal.pmed.0050020. 10.1371/journal.pmed.0050020 PMC2211558 18215107

[15] Schulz KF, Altman DG, Moher D, et al. CONSORT 2010 statement: updated guidelines for reporting parallel group randomised trials. Trials. 2010;11(1):32. 10.1186/1741-7015-8-18. 20334632 10.1186/1745-6215-11-32 PMC2857832

[16] Butcher NJ, Monsour A, Mew EJ, Chan A-W, Moher D, Mayo-Wilson E, Terwee CB, et al. Guidelines for reporting outcomes in trial reports: the CONSORT-outcomes 2022 extension. JAMA. 2022;328(22):2252–64. 10.1001/jama.2022.21022. 36511921 10.1001/jama.2022.21022

[17] Saldanha IJ, Hughes KL, Dodd S, Lasserson T, Kirkham JJ, Wu Y, Lucas SW, Williamson PR. Study found increasing use of core outcome sets in Cochrane systematic reviews and identified facilitators and barriers. J Clin Epidemiol. 2024. 10.1016/j.jclinepi.2024.111277. 38428540 10.1016/j.jclinepi.2024.111277

[18] Wilkinson T, Sinha S, Peek N, Geifman N. Clinical trial data reuse-overcoming complexities in trial design and data sharing. Trials. 2019;20:1–4. 31426840 10.1186/s13063-019-3627-6 PMC6701093

[19] Sanchez-Graillet O, Kramer-Sunderbrink A, Cimiano P. CTrO-Editor: a Web-based Tool to Capture Clinical Trial Data for Aggregation and Pooling. In: Proceedings of the 11th Knowledge Capture Conference. K-CAP ’21, pp. 277–280. Association for Computing Machinery, New York, NY, USA (2021). 10.1145/3460210.3493576.

[20] Wilkinson M, Dumontier M, Aalbersberg I, et al. The FAIR Guiding Principles for scientific data management and stewardship. Sci Data. 2016;3: 160018. 10.1038/sdata.2016.18. 26978244 10.1038/sdata.2016.18 PMC4792175

