# Supplementary figures and images for: Open challenges for the automatic synthesis of clinical trials
Source: BMC Res Notes. 2025 Feb 2;18:50. doi: 10.1186/s13104-025-07121-6 (PMC11789334; doi:10.1186/s13104-025-07121-6)

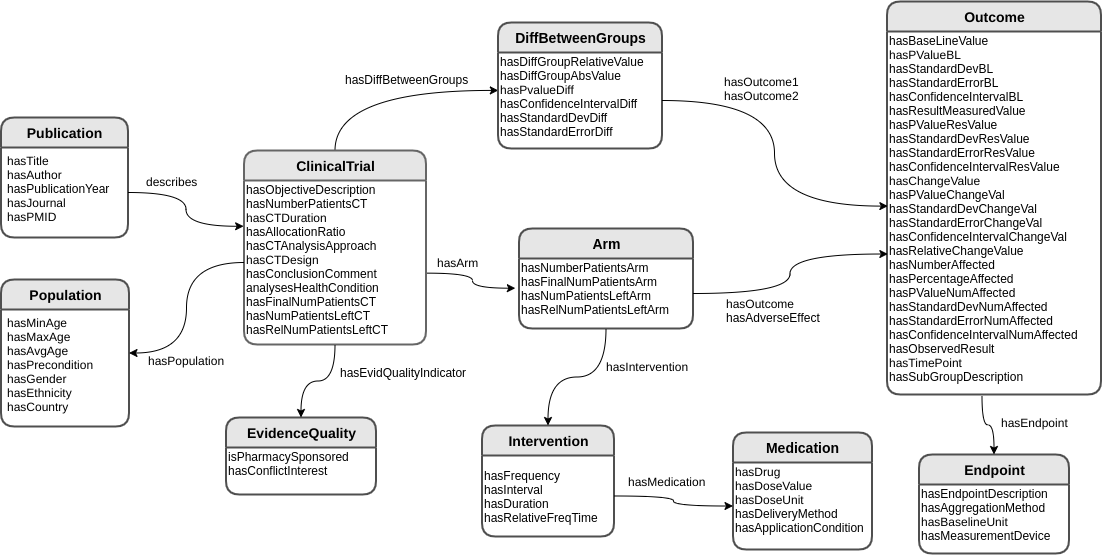

Supplement: Supplementary file 1 — Supplementary Material 1: Supplementary Fig. 1 shows the schema containing the main classes and properties of the C-TrO ontology. This schema was used for the annotation of the CT abstracts, which were used as the dataset for the present study. [file 13104_2025_7121_MOESM1_ESM.png]
